# Effects of grazing on photosynthetic features and soil respiration of rangelands in the Tianshan Mountains of Northwest China

**DOI:** 10.1038/srep30087

**Published:** 2016-07-25

**Authors:** Hua Liu, Runguo Zang, Han Y. H. Chen

**Affiliations:** 1 Department of Forestry and Landscape Architecture, Anhui Agricultural University, Hefei 230036, P. R. China; 2 Faculty of Natural Management, Lakehead University, 955 Oliver Road, Thunder Bay, Ontario P7B 5E1, Canada; 3 Key Laboratory of Forest Ecology and Environment, The State Forestry Administration; Institute of Forest Ecology, Environment and Protection, Chinese Academy of Forestry, Beijing 100091, P. R. China

## Abstract

Rangelands play a critical role in the global carbon cycle. However, the eco-physiological mechanisms associated with the effects of grazing on leaf photosynthesis and soil respiration remain poorly understood. To examine the impacts of grazing on leaf photosynthesis and soil respiration, we measured the photosynthetic parameters of the dominant species (*Trifolium repens*) and the soil respiration in grazed and ungrazed rangelands in the Tianshan Mountains of China. We found that grazing reduced the daily maximum net photosynthetic rate and soil respiration rates by 35% and 15%, respectively. The photosynthetic quantum yield, dark respiratory rate, and water use efficiency of *T. repens* leaves were reduced in grazed plots by 33.3%, 69.2%, and 21.5%, respectively. Our results demonstrated that grazing reduced carbon assimilation while increasing soil respiration within the rangelands in the Tianshan Mountains.

Rangelands contain 20–25% of the global terrestrial carbon within soil and vegetation, and play critical roles in both the global carbon cycle^1^ and in the forage supply for livestock production worldwide^2^. Grazing, however, is considered the key degradation factor in many rangelands of the world, as it results in increased soil and water losses, as well as the degradation of vegetative cover and critical ecosystem services^3^. The effects of grazing on rangelands include the direct degradation of plant and soil^4^ and influences plant biomass and productivity^5^,^6^. Grazers may promote carbon exudation from roots^7^, which leads to a decrease of organic matter in the soil of fragile ecosystems in arid and semiarid regions^8^,^9^.

Grazing may initiate multiple changes that potentially impact eco-physiological mechanisms that are involved in the fixation or loss of carbon through photosynthesis and soil respiration, which are two key features that determine the carbon balance of ecosystems. For example, grazing might alter the warming effects on leaf photosynthesis and dark respiration^10^. Lindwall *et al*.^11^ found that grazing reduced the total carbon content in the leaves of *Bistorta vivipara* by 26%. Chen *et al*.^12^ observed that, following three and five years of grazing exclusion, the net CO_2_ ecosystem exchange of meadow grasslands increased by 47.4% and 15.8%, whereas the ecosystem respiration increased by 33.1% and 4.3%, respectively, in the Tibetan Plateau during the growing season. Moreover, the effects of grazing on net CO_2_ ecosystem exchange appear to be seasonally dependent^13^,^14^. In early spring, grazing has negative effects on grass leaf area and photosynthesis^15^, likely due to the direct damages on plants both above- and below-ground by animals. Han *et al*.^16^ estimated that grazing resulted in a net carbon source of 23.45 g C/m^2^/y in the Xinjiang grasslands.

Bremer *et al*.^17^, Cao *et al*.^18^, and Wang and Fang^19^ all found that grazing reduces the soil respiration, while Wang *et al*.^20^ and Frank *et al*.^21^ reported that grazing accelerates soil respiration. However, Tongway and Ludwig^22^ revealed that soil respiration increases during the process of rangeland recovery. Owensby *et al*.^13^ reported that both grazing exclusion and grazing tall-grass prairie appeared to be carbon-storage neutral, and grazing was not a viable option for increasing carbon sequestration. Jeddi and Chaieb^23^ observed that soil respiration exhibited an increasing trend as the duration of grazing exclusion increased. In a steppe grassland on the Loess Plateau, grazing exclusion markedly increased soil respiration to ~0.36 g C/m^2^/d^24^. These results suggest that the effects of grazing remained debatable.

The majority of grazing studies have employed harvest techniques in the field and laboratory as the methodology for the assessment of grazing effects. Although there have been numerous studies that have examined the effects of grazing on photosynthesis^25^,^26^, plant composition and biomass, biodiversity^27^,^28^ in grasslands worldwide, minimal data on the Tianshan Mountain rangelands is available. The Tianshan ecosystem is a relatively fragile system, which is sensitive to climate change. It also serves as a critical “ecological barrier region” to climate change in Western China and Central Asia^29^. Climatic factors and rangeland management both have potent influences on the seasonal and inter-annual dynamics of carbon fluxes^30^. Grazing mediates the relationships between ecosystem function and carbon flux variability by means of plant physiology^31^. Research gaps, related to grazing effects on the photosynthetic features of plants and soil respiration, constrain the capacity to properly assess the effects of grazing on carbon assimilation and release in arid mountain rangelands. To help address these gaps, we examined the photosynthesis of a dominant plant species and soil respiration in the Tianshan Mountain rangelands under grazed and ungrazed conditions. We hypothesized that 1) plant photosynthetic rate will decrease because of the direct damages on plants by animals, and 2) soil respiration will increase, induced higher soil temperature under grazed conditions. We measured gas exchange and determined light response curves of *T. repens* leaves to assess carbon fixation, and soil respiration rate under grazed and ungrazed conditions in the Tianshan Mountains rangelands.

## Results

### Photosynthetic characteristics

**Figure 1:**
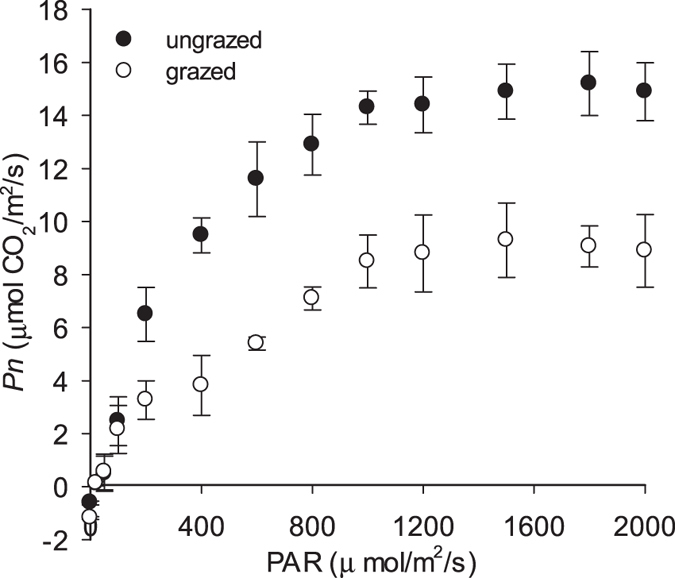
Light response curves of *Trifolum repens* under grazed and ungrazed conditions. The solid hollow dot (○) and solid dot (●) were for grazed and ungrazed, respectively. Vertical bars represent ± SE of the mean (n = 3).

The photosynthetic capacity of *T. repens* leaves under grazed conditions was consistently lower than that of ungrazed conditions across a wide range of photosynthetic active radiation (*PAR*) (Table 1, Fig. 1). In ungrazed plots, light compensation point (*LCP*) was lower, whereas light saturation point (*LSP*) was higher than that in grazed plots. When *PAR* was under 200 μ mol/m^2^/s, the photosynthetic quantum yield (*AQY*) in ungrazed plots was higher than that of the grazed plots. Dark respiration (*R*_*d*_) in ungrazed plots was lower than that of the grazed plots. The maximum photosynthetic rate (*A*_max_) in ungrazed plots was higher than under grazed conditions.

**Figure 2:**
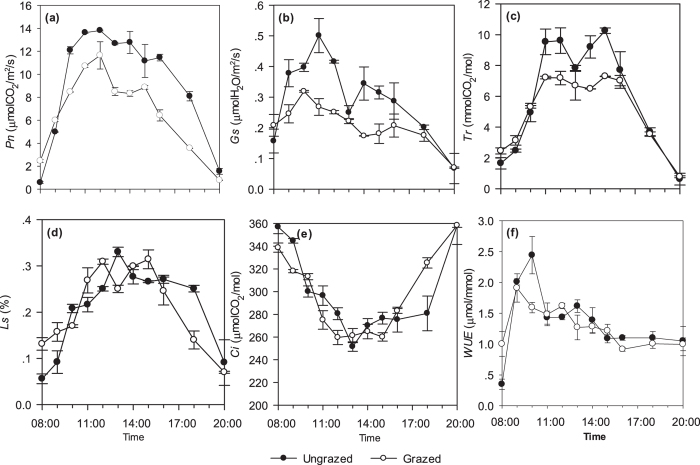
Diurnal changes of the net photosynthetic rate (*Pn)* (**a**), stomata conductance (*Gs*) (**b**), transpiration rate (*Tr*) (**c**), stomata limitation value (*Ls*) (**d**), intercellular CO_2_ concentration (*Ci*) (**e**), and water use efficiency (*WUE*) (**f**) of *Trifolum repens* in ungrazed and grazed plots. The solid hollow dotted lines (—○—) and solid dotted lines (—●—) represented grazed and ungrazed conditions, respectively. Vertical bars represent ± SE of the mean (n = 3 plants).

The diurnal changes of the net photosynthetic rate (*Pn*), stomata conductance (*Gs*) and transpiration rate (*Tr*) were similar, and showed a bimodal pattern (Fig. 2a–c). The *PAR*, ambient air temperature (*T*_*a*_), and blade surface temperature (*T*_*l*_) attained their maximum value at 13:00 hours under daylight. During this period, *Gs* decreased and reduced *Pn* and *Tr*, indicating a midday photosynthetic depression. The daily mean values of *Pn* and *Tr* in grazed plots were significantly lower than in ungrazed plots, *Gs* did not differ significantly between treatments (Table 2). The diurnal changes of the stomata limitation value (*Ls*) peaked at midday under both grazed and ungrazed conditions (Fig. 2d). Diurnal changes in the intercellular CO_2_ concentrations (*Ci*) under both grazed and ungrazed conditions revealed an inverse unimodal pattern (Fig. 2e). The daily mean *Ls* and *Ci* values did not differ significantly between treatments (Table 2). At 8:00 a.m. the water use efficiency (*WUE*) in the grazed plots was higher than that of the ungrazed plots, with both of them being at their lowest levels within a day (Fig. 2f). The initial peak occurred at 9:00 o’clock and 10:00 o’clock, whereas the second peak took place at 12:00 hours and 13:00 hours for ungrazed and grazed conditions, respectively. The daily mean *WUE* values were significantly lower in grazed plots than in ungrazed plots (Table 2).

### Soil respiration

**Figure 3:**
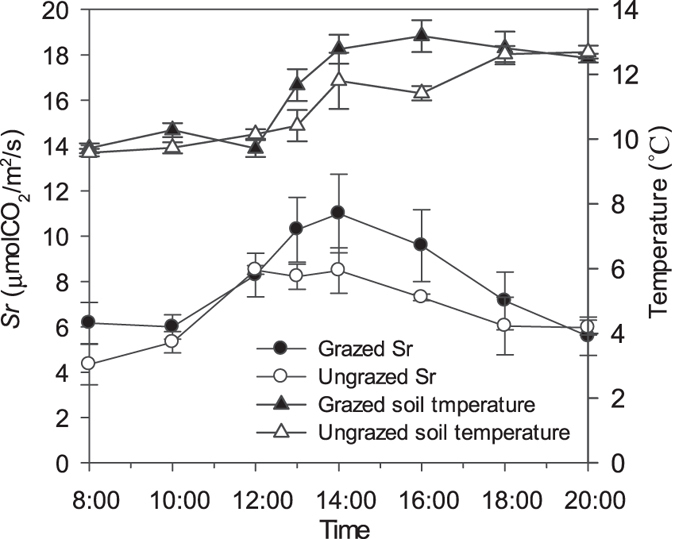
Diurnal changes in the respiratory rate and mean soil temperature of *Trifolum repens*. The solid hollow dotted lines (—○—) and solid dotted lines (—●—) were for soil respiration (*Sr*) in grazed and ungrazed conditions, respectively. The hollow triangular dotted lines (—∆—) and triangular dotted line (—▲—) were for soil temperature in grazed and ungrazed conditions, respectively.

The soil respiration (*Sr*) increased with time during the day while soil temperature peaked during midday in both ungrazed and grazed plots (Fig. 3). *Sr* in ungrazed plots was higher than that of grazed plots between 13:00 and 16:00 hours. The mean value of *Sr* in ungrazed plots (8.01 ± 2.09 μ mol CO_2_/m^2^/s) was significantly higher than in ungrazed plots (6.77 ± 1.58 μ mol CO_2_/m^2^/s) (*p* = 0.017), while the mean values of soil temperature were significantly higher in the grazed than ungrazed plots (*p* = 0.023).

### Correlations of *Pn, Sr* and environmental factors

In the ungrazed plots, there was significant correlation between the *Pn* and ambient air temperature (*T*_*a*_) (*p* < 0.01; r = 0.874), and leaf temperature (*T*_*l*_) (*p* < 0.01; r = 0.880), and *PAR* (*p* < 0.01; r = 0.930). The *Pn* in the grazed plots also had significant correlations with *T*_*a*_(*p* < 0.01; r = 0.742), *T*_*l*_ (*p* < 0.01; r = 0.784), and *PAR* (*p* < 0.01; r = 0.881). Hence, the correlative order among environmental factors with *Pn* was *PAR* > *T*_*l*_ > *T*_*a*_ in both grazed and ungrazed conditions. A multiple regression showed that *Pn* = 3.078 + 0.637 *T*_*a*_ − 0.874 *T*_*l*_ + 0.008 *PAR* across both grazed and ungrazed plots.

The *Sr* of the ungrazed plots was significantly negatively correlated with soil relative humidity (*p* = 0.033; r = −0.797), and was significantly positively correlated with temperature (*p* < 0.01; r = 0.973). In the grazed plots, *Sr* was positively correlated with soil temperature (*p* < 0.01; r = 0.953), but was not with soil relative humidity (*p* = 0.087; r = 0.106).

## Discussion

Our results revealed that *T. repens* in ungrazed conditions had higher adaptability to the light environment than in grazed conditions, which are similar to previous results^32^,^33^, suggesting that plants under ungrazed conditions are more capable of making use of light for carbon assimilation. Moreover, *T. repens* under ungrazed conditions also had lower dark respiration rate than under grazed conditions compared with ungrazed conditions, the net carbon assimilation (as indicated by the daily mean of net photosynthetic rate) for *T. Repens* under grazed conditions was decreased by 15.4%. Our findings were consistent with, albeit with a higher value than, the results reported by Lindwall *et al*.^11^ and Han *et al*.^16^. This was contrary to the reports that grazing may increase the photosynthetic capacity of the leaves in the first two years of fencing that grew in meadow grasslands on the Tibetan Plateau^34^, and have a greater photosynthetic capacity in grazing desert steppe which caused by suitable environmental conditions and longer growing time in growing period^35^.

The midday depression of photosynthesis comprises a self-regulating ecological adaptation of plants that corresponds to carbon exchange^36^. Regardless of the grazing condition, the net photosynthetic rate, stomata conductance, and transpiration rate fit a bimodal pattern for *T. repens* (Fig. 2a–c). An adaptation of the leaves of practically all mesophytes and xerophytes through the closing of their stomata^37^ could avoid water loss at noon, which reduces carbon uptake^38^. The midday depression of the net photosynthetic rate of *T. repens* in ungrazed and grazed situations occurred at 13:00 hours and 14:00 hours, respectively. The values of the net photosynthetic rate of *T. repens* leaves under grazed conditions may be more markedly depressed than those under ungrazed conditions after 10:00 o’clock (Fig. 2a). Typically, heterogeneous stomatal behaviors have been employed to calculate leaf conductance from water vapor exchange, which has variable effects on the photosynthesis of plants^39^, where grazing can depress stomatal conductance (Fig. 2b). The decline in stomatal conductance might reduce excess water vapor loss directly through boundary obstacles and stomatal closure^40^, and the transpiration rate had synchronous changes with the net photosynthetic rate and the stomata conductance (Fig. 2c).

Optimal stomatal behavior has been influential in explaining how carbon gain and water loss are balanced, based on the hypothesis that plants regulate stomatal opening and closing in such a way as to maximize (A − λ E), where A is photosynthesis, E is transpiration, and λ is the marginal carbon cost of water to the plant^41^. Hence, the use of the term stomatal limitation, including stomatal limitation and non-stomatal limitation to refer to this idea, may explain the phenomenon of the midday depression of photosynthesis^42^. Farquhar and Sharkey^43^ considered that when the net photosynthetic rate and intercellular CO_2_ concentrations changed in the same direction, both of them were diminished, where only the stomatal limitation value was increased. The net photosynthetic rate may be thought to be caused by stomata factors, or via the decline of mesophyll cell carboxylation activity. In this paper, the net photosynthetic rate and intercellular CO_2_ concentration of *T. repens* declined from 12:00~13:00 hours in ungrazed plots, and the stomatal limitation value increased (Fig. 2d). These values indicated that the midday depression of photosynthesis during this period of time was initiated by stomatal limitation, while non-stomatal limitation presided in the grazed plots. One reason was that grazing impacted soil conditions, which caused the water potential in root systems to be altered; thereby affecting the stomata characteristics, such as stomata opening. An additional factor was that the leaves in grazed plots were typically younger (following grazing and renewal) than those in ungrazed plots. Their photosynthetic abilities were not stable, and total photosynthetic capacity was lower, which was induced by their light and temperature enduring ability, *Rubp* carboxylase activity, and their net photosynthetic rate was reduced^44^. These results considered that the maximum quantum efficiency of PSII photochemistry (F(v)/F(m)) increased continuously, from younger leaves to fully mature leaves, and suggested that mature leaves had the capacity to recover more quickly from photo-inhibition than did younger leaves. Further, the ratio of intercellular CO_2_ and ambient air CO_2_ concentrations in the grazed plots was higher than that of ungrazed plots at 13:00 hours. This revealed that the stomata were opening during the course of photosynthesis in the grazed plots; however, the net photosynthetic rate value declined. As a result, we propose that the dominant effect was carboxylase activity, whereas the mesophyll cell stomata regulation functions were secondary^44^,^45^. When leaf conductance to CO_2_ was high and CO_2_ concentrations in the intercellular spaces (Fig. 2e) were being continually drawn down by the rapid fixation of carbon, the CO_2_ influx from the ambient atmosphere surrounding the leaf will subsequently be high. This offered a good explanation for how the transpiration rate is affected by vapor tension differences between the leaves and ambient air, and that the transpiration rate decline induced by stomata conductance was decreased in the field. Water use efficiency provided the best index for measuring the ratio of carbon fixation, water consumption, and to evaluate plant adaptability under stressed conditions^46^, which was observed to be higher in the morning than that in the afternoon in both conditions (Fig. 2f). Grazing decreased LAI and contribution on the microclimate of plant layer caused the mean daily *WUE* under ungrazed conditions was significant higher than that under grazed^47^.

Our study indicated that grazing increased soil respiration significantly, and soil CO_2_ fluxes in grazed plots were 2.69–29.63% higher than those in the ungrazed plots at different times of the day (Fig. 3), leading to a daily average of 15% higher soil respiration in the grazed than ungrazed plots. The respiratory rate of the soil is determined by climate, particularly temperature, water, and their interactions^48^. We sought to elucidate how these factors influenced soil respiration. Keith *et al*.^49^ proposed that 97% of the variances in soil respiration may be explained by temperature and moisture. Our results indicated that diurnal change patterns in soil respiratory rates were similar in both ungrazed and grazed plots, and that there were significant positive correlations between soil respiration and soil temperature in both ungrazed and grazed plots, with the former coefficient being higher than latter. There was a significant negative correlation between soil respiration and soil humidity in ungrazed plots, which was consistent with the results of Conant *et al*.^50^, who reported that soil respiration increases with reduced soil humidity. In this study, there was no significant relationship found between soil respiration and soil humidity in grazed plots. An additional factor that was considered by Tanentzap and Coomes^51^ included that grazing may have variable effects on carbon storage in soil through the influence of herbivores on litter decomposition and nitrogen, which will require further study in the future.

## Conclusion

*T. Repens* exhibited higher light utilization capacities in ungrazed plots in contrast to grazed plots, as grazing depressed the net photosynthetic rates of *T. repens* leaves. Soil respiration rates were lower under ungrazed conditions than for grazed conditions. *T. repens* leaves possessed stomatal or non-stomatal limitationsin order to facilitate acclimatization to the effects of grazing and environmental factors. Grazing should be recognized as a critical influencing factor toward the evaluation of carbon balance and its global change effects on rangeland ecosystems that are dominated by *T. repens* in the Tianshan Mountains, in Northwest China.

## Materials and Methods

### Study area

The research for this study was conducted at the Tianshan Forest Ecosystem Observation and Research Station, State Forest Administration (N 43°09′~43°28′, E 87°12′~87°50′), which is located in the Tianshan Mountains of Central Asia, Xinjiang Uygur Autonomous Region of China. The study area was 3690 km^2^, with the elevation ranging from 1908 m to 2017 m, and a mean slope of 39°. The climate is temperate continental, with an annual frost-free period of 150~160 d, average annual precipitation of 410 mm, with highest and lowest temperatures of 30 °C and −38 °C, respectively, and an annual mean temperature of 5 °C. The dominant soil is grey forest and the dominant herbage at different elevations of mountain rangeland includes *Trifolium repens*, *Achillea millefolium*, *Aegopodium podagraria*, *Alchemila tianschanica*, and *Poa nemoralis*.

### Sampling design

The elevation of our measuring plots was1956 m~1983 m, and the mean total vegetation coverage was 90%. Three 1.4 ha ungrazed plots were fenced in 2013 to exclude domestic grazing animals, whereas the grazed plots formed three 1 ha portions of rangeland adjacent to the ungrazed plots, and free grazing was adopted all year round with the grazing intensity of one sheep per hectare. Three grazed subplots and three ungrazed subplots (the dimensions of each plot was 5 × 5 m) were randomly selected. The *T. repens* coverage in these two types of plots ranged from 71 to 75%, and from 77 to 82%, respectively. In the grazed subplots, every selected plant was protected by small fences to ensure that it had sound leaves for measurement of photosynthetic parameters. In the selection of *T. repens* for measurements, three replicates were randomly assigned for each plot; i.e., three plants were randomly selected in each plot, and three portions of trefoil leaves, which had been grown under full sun, were randomly selected and marked for the measurement of photosynthetic and related environmental parameters.

### Photosynthetic response to light

A portable photosynthesis system with a LED light resource (LI-6400-02B, LI-COR Inc. Lincoln, NE, USA) was employed to measure the photosynthetic response to variable light. Light response curves were generated automatically by measuring the net photosynthetic rate (*Pn*) of *T. repens* at steady state under different levels of photosynthetic active radiation (*PAR*) (0~2000 μ mol/m^2^/s), beginning with 2000 μ mol/m^2^/s. The *PAR* was decreased stepwise via the illumination gradient of 2000, 1800, 1600, 1400, 1200, 1000, 800, 600, 400, 200, 100, 50, 20, and 0 μ mol/m^2^/s. These measurements were conducted under an ambient air temperature (*T*_*a*_) of 20.49 ± 0.22 °C, with a mean leaf temperature (*T*_*l*_) of 19.54 ± 0.88 °C, ambient air CO_2_ density of 375.75 ± 1.06μ mol CO_2_/mol, with a mean relative humidity of 37.64 ± 1.25%. The study was performed from 10:00 a.m. to 12:00 p.m. on September 5, 7, and 9, 2015. The relationship between *PAR* and *Pn*, light compensation point (*LCP*) and light saturation point (*LSP*) were automatically obtained from the curve. Simultaneously, the apparent quantum requirement (*AQY*) could be found from the slope of the regression curve that showed the relationship between the net photosynthetic rate and *PAR* measured in 200, 100, 50, 20, and 0 μ mol/m^2^/s. The intercept with the vertical axes was the dark respiration rate (*R*_*d*_).

### Diurnal changeof *Pn*

The *Pn* of *T. repens* grown under grazed and ungrazed conditions were measured using portable photosynthesis system (LI-cor 6400-02, LI-COR Inc., Lincoln, NE, USA) equipment with an infrared CO_2_/H_2_O analyzer over three clear days in September 6, 8, and 10, 2015. The leaf gas-exchange of diurnal curves was run each day, with measurements made from 8:00 to 20:00 hours at 1 h intervals. In each instance, three segments of leaf replicates were utilized. Additional gas exchange indexes including the transpiration rate (*Tr*), stomata conductance (*Gs*), intercellular CO_2_ concentration (*Ci*), and stomata limitation value (*Ls*) were measured. Simultaneously, environmental indexes, including *PAR*, ambient air temperature (*T*_*a*_), leaf surface temperature (*T*_*l*_), and relative humidity (*RH*) were also measured with *Pn*. Equations for the calculation of water use efficiency (*WUE*), and *Ls* were as follows:


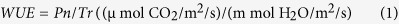



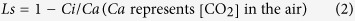


The leaves of *T. repens*are palm shaped having three multifoliage segments, with each single leaf area being less than 6 cm^2^ (the standard leaf chamber area was 6 cm^2^ of Li-6400). As such, one segment of trefoil leaves was selected and marked for every measurement, after which the areas were measured using leaf area meter (Li-cor 3100, LI-COR Inc., Lincoln, NE, USA). The value of each leaf area was input as a measurement parameter for an Area Module, which matched up in correspondence to *T. repens*.

### Soil respiratory rate measurement

Respiration rates of the soil (*Sr*) under the rangelands dominated by *T. repens* were measured using an IRGA (LI-6400-09, LI-COR Inc., Lincoln, NE, USA), which was connected to a portable photosynthesis system (LI-cor 6400, LI-COR Inc., Lincoln, NE, USA) over three clear days in September12, 13, and 15, 2015. Three soil circle collars created by a PVC tube were randomly buried in each ungrazed and grazed plot, respectively. The height of each collar was 5.0 cm, with an inner diameter of 11.0 cm, and a 5.0 mm wall thickness. Each of the collars had a soil area of 80.0 cm^2^, and the soil was 3.0 cm deep. Prior to measurements, the living plants were cut off, and all of the collars were introduced into the soil for 24 hours. Measurements were made on three replicate soil collars, and the soil temperatures at 10 cm depth were recorded for each instance using a Li-6400 soil temperature probe. Soil respiration rate measurements were made in each collar between 8:00 and 20:00 hours.

### Data analysis

We used the analysis of variance (ANOVA) to test the effects of grazing on the photosynthesis indexes and soil respiration rate. To achieve a mechanistic understanding of the changes in photosynthesis, we tested how grazing and environmental factors affected the gas exchange indexes, and assessed the associations between these variables with the net photosynthetic rate by Pearson correlation and regression analysis. All statistical analyses were performed using the SPSS version 21 software package (SPSS Inc., Chicago, IL, USA).

## Additional Information

**How to cite this article**: Liu, H. *et al*. Effects of grazing on photosynthetic features and soil respiration of rangelands in the Tianshan Mountains of Northwest China. *Sci. Rep.* **6**, 30087; doi: 10.1038/srep30087 (2016).

## Figures and Tables

**Table 1:** Photosynthetic parameters of *Trifolium repens* grown in ungrazed and grazed plots (Mean ± 1 s.e.m.).

Parameter	Grazed	Ungrazed
Light compensation point (μ mol/m^2^/s)	12.90 ± 1.02^a^	8.15 ± 0.17^b^
Light saturation point (μ mol/m^2^/s)	1500 ± 2.95^a^	1800 ± 3.00^b^
Dark respiratory rate (μ mol O_2_/m^2^/s)	0.52 ± 0.13^a^	0.16 ± 0.04^b^
Photosynthetic quantum yield (μ mol/m^2^/s)	0.02 ± 0.00^a^	0.03 ± 0.00^b^
Maximum photosynthetic rate (μ mol CO_2_/m^2^/s)	9.30 ± 0.49^a^	15.20 ± 0.81^b^

Different uppercase letters indicate significant difference between grazed and ungrazed treatments at α = 0.05.

**Table 2:** The daily mean values of net photosynthetic rate, stomata conductance, transpiration rate, stomata limitation value, intercellular CO_2_ concentration and water use efficiency of *Trifolum repens* leaves in ungrazed and grazed plots (Mean ± 1s.e.m.).

Parameter	Grazed	Ungrazed
Net photosynthetic rate (μ mol CO_2_/m^2^/s)	6.89 ± 1.03^a^	9.35 ± 1.46^b^
Stomata conductance (μ mol H_2_O/m^2^/s)	0.21 ± 0.01^a^	0.30 ± 0.04^a^
Transpiration rate (m mol CO_2_/mol)	5.19 ± 0.69^a^	6.14 ± 1.07^b^
Stomata limitation value (%)	0.21 ± 0.02^a^	0.21 ± 0.02^a^
Intercellular CO_2_ concentration (μ mol CO_2_/mol)	296.13 ± 10.77^a^	299.26 ± 11.19^a^
Water use efficiency (μ mol/m mol)	1.29 ± 0.03^a^	1.36 ± 0.05^b^

Different uppercase letters indicate significant difference between grazed and ungrazed treatments at α = 0.05.
