# Corrigendum: Effects of grazing on photosynthetic features and soil respiration of rangelands in the Tianshan Mountains of Northwest China

**DOI:** 10.1038/srep33937

**Published:** 2016-09-30

**Authors:** Hua Liu, Runguo Zang, Han Y. H. Chen

Scientific Reports 6: Article number: 3008710.1038/srep30087; published online: 07 25 2016; updated: 09 30 2016

The original version of this Article contained typographical errors. In the Abstract,

“We found that grazing reduced the daily maximum net photosynthetic rate and soil respiration rates by 35% and 15%, respectively”.

now reads:

“We found that grazing reduced the daily maximum net photosynthetic rate and increased soil respiration rates by 35% and 15%, respectively”.

In the Results section under sub-heading ‘Soil respiration’,

“The soil respiration (*Sr*) increased with time during the day while soil temperature peaked during midday in both ungrazed and grazed plots (Fig. 3). *Sr* in ungrazed plots was higher than that of grazed plots between 13:00 and 16:00 hours. The mean value of *Sr* in ungrazed plots (8.01 ± 2.09 μ mol CO_2_/m^2^/s) was significantly higher than in ungrazed plots (6.77 ± 1.58 μ mol CO_2_/m^2^/s) (*p* = 0.017), while the mean values of soil temperature were significantly higher in the grazed than ungrazed plots (*p* = 0.023)”.

now reads:

“The soil respiration (*Sr*) increased with time during the day while soil temperature peaked during midday in both ungrazed and grazed plots (Fig. 3). *Sr* in grazed plots was higher than that of ungrazed plots between 13:00 and 16:00 hours. The mean value of *Sr* in grazed plots (8.01 ± 2.09 μ mol CO_2_/m^2^/s) was significantly higher than in ungrazed plots (6.77 ± 1.58 μ mol CO_2_/m^2^/s) (*p* = 0.017), while the mean values of soil temperature were significantly higher in the grazed than ungrazed plots (*p* = 0.023)”.

These errors have now been corrected in the PDF and HTML versions of the Article.

